# Exploring Appropriate Strategies for Vaccination against Classical Swine Fever under a Dynamic Change in Antibody Titer in Sows after Starting Vaccination in a Japanese Farm Setting

**DOI:** 10.1155/2023/5541976

**Published:** 2023-11-30

**Authors:** Makoto Ukita, Keisuke Kuwata, Eiji Tanaka, Ryota Matsuyama, Norikazu Isoda, Yoshihiro Sakoda, Takehisa Yamamoto, Kohei Makita

**Affiliations:** ^1^Veterinary Epidemiology Unit, Graduate School of Veterinary Medicine, Rakuno Gakuen University, 582 Bunkyodai Midorimachi, Ebetsu, Hokkaido 069–8501, Japan; ^2^Gifu Prefecture Central Livestock Hygiene Service Center, 1–1 Yanagito, Gifu 501–1112, Japan; ^3^Livestock Epidemic Prevention and Control Division, Department of Agricultural Policy, Gifu Prefectural Government, 2–1-1 Minami-Yabuta, Gifu 500–8570, Japan; ^4^Laboratory of Microbiology, Department of Disease Control, Faculty of Veterinary Medicine, Hokkaido University, Kita 18, Nishi 9, Kita-Ku, Sapporo, Hokkaido 060–0818, Japan; ^5^Viral Disease and Epidemiology Research Division, National Institute of Animal Health, National Agriculture Research Organization, 3–1-5 Kannondai, Tsukuba, Ibaraki 305–0856, Japan

## Abstract

After 26 years of absence in Japan, a classical swine fever (CSF) outbreak occurred at a domestic pig farm in 2018. Vaccination against the CSF virus with a live attenuated vaccine at pig farms was restarted in October 2019, which was 13 years after the 2006 ban on vaccination. An individual-based simulation model for CSF antibody dynamics was developed to determine an effective CSF vaccination strategy for pig populations. In creating a simulated pig herd, the optimal vaccination age of piglets and the effect of vaccinating piglets twice were evaluated. Additionally, the herd immunity was monitored every 6 months for 4 years after the start of vaccination, and the effects of intensive sow replacement policies were assessed. The simulation results indicated that the vaccination age should be delayed relative to the age used before the 2006 ban on vaccination and shifted earlier, from 8 weeks to 6 weeks, as time elapses. The simulations indicated a tradeoff in protection between the weaning period (i.e., maternally derived antibodies) and the fattening period (i.e., by vaccine-induced antibodies). Mixing sows with high and low antibody titers, particularly sows that received the first vaccination and those born after the start of vaccination, resulted in a high variation in antibody titer among pigs on the farm. This study also clarified the positive effect of intensive sow replacement strategies on shortening the period in which sows show diverse titers. Differences in sow replacement rates among farms and/or the time lag in starting vaccination in different prefectures result in heterogeneity in herd immunity in Japan; thus, herd immunity status should be examined at every farm using this simulation model.

## 1. Introduction

Classical swine fever (CSF) is a highly contagious viral disease affecting domestic pigs and wild boars [1]. The disease is caused by the CSF virus (CSFV), which belongs to the family *Flaviviridae*, genus *Pestivirus*. CSFV is classified into three genotypes (1, 2, and 3) with several subgenotypes (1.1–1.4, 2.1–2.3, and 3.1–3.4) [2, 3]. CSF has a significant socioeconomic impact on swine production due to the high virulence of CSFV; therefore, the disease occurrence must be notified to the World Organisation for Animal Health (WOAH, founded as OIE) [4]. Most of the Japanese pig farming is for industrial purposes. The average number of pigs raised per farm was 2,493, and on farrow-to-finisher or breeding farms, the average number of sows per farm was 287 in 2022 [5]. Since an outbreak of CSF requires the culling of the entire herd, economic losses are substantial.

**Figure 1:**
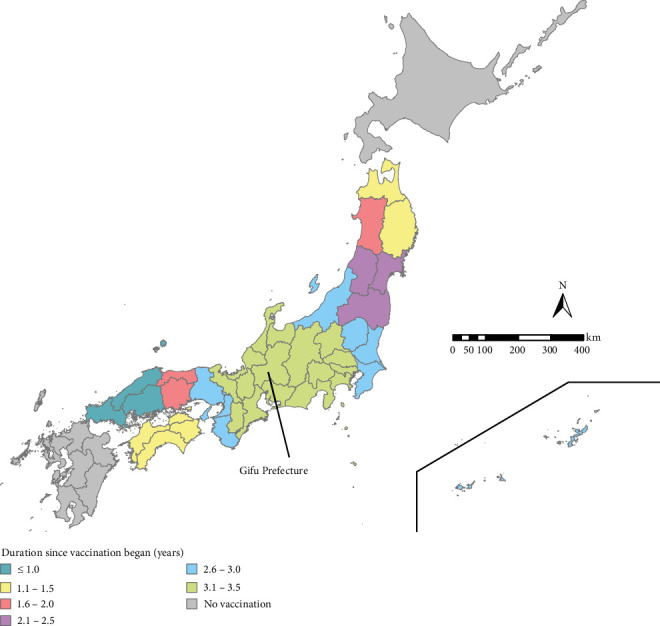
Elapsed time since the start of CSF vaccination in prefectures in Japan (as of March 31, 2023).

In 1969, an attenuated live vaccine for CSF (GPE^−^) was developed from a highly virulent genotype-1 CSFV strain (ALD strain) [6]. After the introduction of this vaccine, the frequency of CSF outbreaks on domestic swine farms in Japan decreased dramatically [7]. In 2000, after an 8-year absence of CSF outbreaks, the use of the GPE^−^ vaccine for pigs was restricted, and the use of the vaccine was completely banned in 2006. Japan was officially recognized as a CSF-free country in 2015 by the OIE (at that time) [8]. However, in September 2018, the first outbreak of CSF since 1992 occurred on a pig farm in Gifu Prefecture, located in the central part of Japan, and this outbreak reportedly involved a CSFV of subgenotype 2.1b [9, 10]. The results of genetic analysis strongly suggested the reintroduction of CSFV from outside of Japan [10]. Soon after the first CSF case was confirmed on the farm, CSFV infection was found to have spread to wild boars. In conjunction with the progression of CSFV infection in wild boars, a series of outbreaks occurred on pig farms in Gifu and neighboring prefectures. To prevent the further spread of CSF among wild boars, an oral bait vaccination program was initiated in March 2019 [11]. Although veterinary authorities made efforts to strengthen biosecurity measures on farms, farm outbreaks continued. Finally, preventive vaccination at pig farms using the GPE^−^ vaccine was reinitiated in Gifu and the nine adjacent prefectures in October 2019 [12]. A total of 39 of 47 Japanese prefectures have vaccinated domestic pigs as of March 31, 2023 (Figure 1).

The national-level CSF vaccination program is managed by the Ministry of Agriculture, Forestry, and Fisheries (MAFF), and prefectures are required to develop their prefectural-level vaccination program according to the Guideline to Control CSF provided by the MAFF [13]. According to this guideline, all pigs on farms in the prefectures designated by MAFF as within the vaccination area (except pigs within 20 days of slaughter or suckling piglets) should receive an initial vaccination. Subsequently, newborn piglets should be vaccinated at 1–2 months of age, and sow candidates are additionally vaccinated 6 months after their first vaccination. As pigs mature to breeding sows, they are vaccinated yearly, with a maximum of four doses over the lifespan.

**Figure 2:**
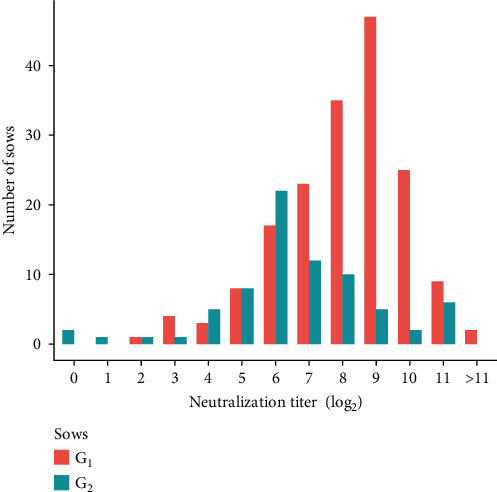
Neutralization titer of G_1_ (*n* = 166) and G_2_ (*n* = 83) sows sampled in Gifu Prefecture between May and August 2021 (Gifu Prefecture, personal communication).

The GPE^−^ vaccine is a live-attenuated vaccine that interferes with the immune response of piglets if the maternally derived antibody (MDA) level is high; thus, the timing of vaccination is critically important in achieving the herd immunity threshold. Before the complete ban on the CSF vaccination in 2006, the GPE^−^ vaccine was administered to pigs at 35–42 days of age [14]. After the CSF vaccination was restarted in 2019, sows that were vaccinated for the first time (first generation: G_1_) did not have MDAs; thus, they exhibited higher antibody titers compared with before the 2006 vaccination ban. As a result, piglets born to G_1_ sows (second generation: G_2_) received high MDA titers through the colostrum [15], and the high levels of preexisting MDAs interfered with the immune response of G_2_ piglets at the vaccination age. To avoid this interference, the vaccination age of G_2_ piglets can be delayed beyond the prescribed vaccination age. However, delaying vaccination carries a risk of CSF infection among piglets due to insufficient protective immunity resulting from a lower MDA titer in the herd. Moreover, regarding the 2021 situation, a serological survey showed that G_2_ sows exhibited lower antibody levels than G_1_ sows, suggesting interference of the immune response against the vaccine (Gifu Prefecture, personal communication, Figure 2). Therefore, a practical scheme for determining the “best-bet” vaccination age for piglets is critically needed.

In addition to the high MDA titer received by the G_1_ sows, another problem became apparent: high variation in antibody titer distribution among sows (Gifu Prefecture, personal communication). On farms, breeding sows are replaced to maintain reproductive performance. Soon after the start of vaccination, the sow herd consists of only G_1_ sows, and then G_1_ sows are gradually culled and replaced by G_2_ and subsequent generations. The mixture of G_1_ and subsequent generations can result in wide variation in antibody titer. Accordingly, the offspring can also exhibit wide variation in MDA titer, making it difficult to determine the appropriate vaccination age for piglets. Sow replacement strategies that rapidly reduce variation in titer among sows and their offspring should be considered.

In this study, an individual-based model to predict the decline in MDAs and the increase in vaccine-induced antibodies (VIAs) was established using data from serological tests conducted on pigs on vaccinated farms. This model involves the nonparametric bootstrap resampling of the actual data on the difference in antibody titers between sows and their piglets and antibody dynamics after vaccination. Performing simulations with this model, we aimed to determine the appropriate vaccination strategy. To achieve this aim, we focused on three parameters: the vaccination age of piglets, the number of vaccination shots administered to fattening pigs, and the method of sow replacement. This model was also intended to be developed for the use of the selection of vaccination age at any size of pig farms during the course of vaccination in Japan.

## 2. Materials and Methods

### 2.1. Model Structure

A simulation model that considers a farrow-to-finisher farm was developed. A simulation of 30 sows randomly selected from the observed 168 sows that predicted the immune response of their litters on a farm was iterated 100 times. The sample size of 30 was based on practical considerations, as it is a realistic manageable number to collect blood samples in a farm by veterinarians in prefectural Livestock Hygiene Service Centers (LHSCs) and the minimum number at which the central limit theorem starts to hold [16]. Bootstrap resampling from a small number of samples is known to successfully achieve an estimation of the mean of a mother population [17], regardless of the size of a farm. Also, estimating the appropriate vaccination age using this simulation model requires intensive calculation, and starting with 30 sows can avoid excessive computational burden. On the model farm, each sow bears 12 piglets. Sows farrow for the first time at 12 months of age and subsequently farrow every 6 months. Forty percent of sows are replaced each year as a default setting based on the personal communication with Japanese swine veterinarians (but this is increased to 60% in “intensive replacement,” see Section 2.10). Finisher pigs are slaughtered at 6 months of age, and the farm does not buy-in animals from outside.

**Figure 3:**
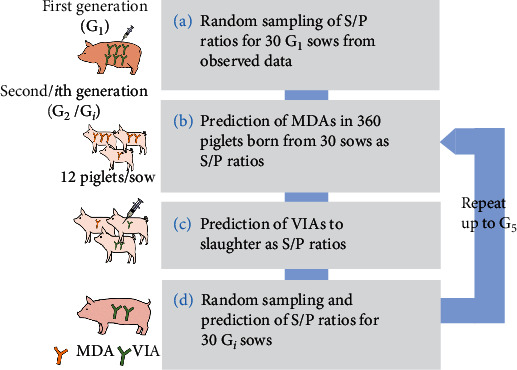
Scheme for simulating antibody dynamics among different generations of pigs: (a) the antibody titers of the first generation of sows (G_1_, 30 individuals) in our simulation were determined by random sampling of the observed S/P ratio data; (b) the titers of maternally derived antibodies (MDAs) of the second generation (G_2_, 360 animals; 12 from each G_1_ sow) were predicted from the antibody titers of the G_1_ sows; (c) the vaccine-induced antibody (VIA) titers until the slaughter age (26 weeks) were predicted; (d) 30 sow candidates were randomly resampled as sows for the next generation. The antibody titers of G_3_ were predicted based on those of G_2_, and the prediction was repeated up to G_5_.

Figure 3 illustrates a simulation scheme for antibody dynamics among different generations of pigs. First, 30 random samples of G_1_ were taken from actual field data consisting of anti-CSFV antibody titer values from 168 G_1_ sows at farrowing (Figure 3(a)). As the LHSCs in Japan primarily use the sample to positive (S/P) ratio calculated from the results of enzyme-linked immunosorbent assay (ELISA) data (Classical Swine Fever ELISA kit II, JNC Corp., Tokyo, Japan), the S/P ratio was used in our analysis. Based on the S/P ratio for sows on the date of delivery, the decline in MDAs in piglets until the date of vaccination was predicted (Figure 3(b)). Depending on the level of MDAs at the vaccination age, subsequent VIAs were predicted (Figure 3(c)). Thirty animals were randomly resampled from the pool of sow candidates vaccinated at 6 months after the first vaccination (Figure 3(d)). Based on their antibody titers, the antibody dynamics of the next generation were predicted. The predictions shown in (Figure 3(b)–3(d)) were repeatedly carried out up to the fifth generation.

### 2.2. Collection of CSF Diagnosis Data

This study used field data for serum samples from domestic pigs subjected to CSF vaccination conducted by the LHSCs in Aichi and Gifu Prefectures. In addition, the test results of consecutive blood sampling after vaccination conducted on seven anonymized farms were also included (Table 1). Seven datasets were used to (1) estimate the model parameters for interconverting neutralization titers and S/P ratios, (2) predict neutralization titers of 1-day-old piglets from neutralization titers of their sows, (3) predict declines in neutralization titer of MDAs, (4) sample the S/P ratio distribution of G_1_ sows, (5) predict changes in VIAs, (6) calculate S/P ratio cutoff values for log_2_ neutralization titer, and (7) validate the prediction of antibody titers among fattening pigs.

### 2.3. Interconversion Model for Neutralization Titers and S/P Ratios

The decline in MDAs among piglets was predicted by (i) converting the S/P ratio to neutralization titer, (ii) predicting the decline using neutralization titer, and (iii) reconverting the declined neutralization titer to S/P ratio. This is because antibody titers are primarily measured based on the S/P ratio by an ELISA in the field; however, a method to predict the decline of MDAs in the S/P ratio has not been established. The neutralization titer, by contrast, is known to decline constantly on a logarithmic scale based on the concept of antibody half-life [18]. Therefore, an interconversion model for neutralization titers and S/P ratios was developed to predict MDAs in piglets on a given day after delivery. From the definition of the S/P ratio [19], the numerator (i.e., the difference in optical absorbance between samples with and without the antigen) can be regarded as the proportion of antigen coating the wells that are bound by antibody in the sample serum (hereafter, the proportion *P*_*s*_). In contrast, the denominator (i.e., the difference in optical absorbance between controls with and without the antigen) can be regarded as the proportion of antigen coating the wells that are bound by the positive control antibody (hereinafter, the proportion *P*_*c*_). We assumed that both *P*_*s*_ and *P*_*c*_ can be described by a logistic function, and their linear predictors can be written by the logarithm of the neutralization titer as explanatory variables, as follows in Equations (1)–(3):

(1) $$\begin{array}{c} SP_{s}=\frac{P_{s}}{P_{c}}=\frac{\text{logistic}\left(Y_{s}\right)}{\text{logistic}\left(Y_{c}\right)}, \end{array}$$

(2) $$\begin{array}{c} Y_{s}=\log\frac{P_{s}}{1-P_{s}}=\beta +a\log_{2}\left(NT_{s}\right), \end{array}$$

and

(3) $$\begin{array}{c} Y_{c}=\log\frac{P_{c}}{1-P_{c}}=\beta +a\log_{2}\left(NT_{c}\right), \end{array}$$

where *SP*_*s*_ represents the sample serum S/P ratio, *Y*_*s*_ represents the logit of *P*_*s*_, *Y*_*c*_ the logit of *P*_*c*_, *β* represents the intercept of the logistic equation, *a* represents the slope of the logistic equation, *NT*_*s*_ represents the sample serum neutralization titer, and *NT*_*c*_ represents the positive control neutralization titer. *NT*_*c*_ was assumed to be constant. *Supplementary 1* shows the relationship between the paired neutralization titer and S/P ratio for all types of pigs on seven farms in five prefectures sampled between May 2020 and December 2021. Using 1,160 paired samples of S/P ratio (*SP*_*s*_) and *NT*_*s*_, *NT*_*c*_, *β*, and *a* were estimated using the least squares method. *SP*_*s*_ can then be calculated in the field using these estimated parameters and neutralization titers. In this estimation, paired samples with high neutralization titers (titer of 2,048- and 4,096-fold (i.e., 11 and 12 log_2_ titer, respectively)) were excluded due to the small number of highly variable samples (*n* = 14 and 7, respectively).

The S/P ratios converted to neutralization titers above 12 log_2_ or the predicted level at the complete binding of all of the antigen coating the wells were replaced with randomly selected S/P ratio values from the pool of paired observed neutralization titers with *S*/*P* ratios greater than the threshold to improve the predictability of neutralization titers for high S/P ratios. Negative values for the logarithm of neutralization titers were assumed to be zero. The validity of the model was evaluated by comparing the distributions of observed and converted neutralization titers from observed S/P ratios using the Kolmogorov–Smirnov test.

### 2.4. Predicting Piglet Acquisition of MDAs from Sows

In our simulation, the titers of MDAs among agent piglets (i.e., the individual animals simulated) at 1 day of age were predicted based on the differences in neutralization titers between sows and their litters. To obtain the pool of the observed differences, paired neutralization titers were collected from 63 piglets and their 9 respective sows (Table 1). As we observed that the distribution of the difference in antibody titer between litters and their sows differed based on the sows' antibody titer, sows were classified into three groups based on the level of neutralization titer: <8 log_2_, 8–10 log_2_, and >10 log_2_. In accordance with this grouping, the observed differences in the logarithm of neutralization titer between sows at delivery (within 1 month before farrowing) and their litters at 1 day of age were pooled in each group. Using these pooled data, MDA titers among piglet agents at 1 day of age were predicted using the following procedure: (i) sow S/P ratios were converted to neutralization titers using the model for interconverting S/P ratios to neutralization titers, (ii) sows were classified into three groups according to the neutralization titer (i.e., <8 log_2_, 8–10 log_2_, and >10 log_2_), (iii) a difference was randomly sampled from the pool of each group, and (iv) the MDA titer of a litter at 1 day of age was calculated by subtracting the sampled difference from the neutralization titer of the litter's sow.

### 2.5. Predicting the Decline in MDAs

The decline in MDAs was predicted based on the linearity of log_2_ (neutralization titer). To estimate the slope of the decline in MDAs, data tracing the neutralization titers of 386 piglets were used (Table 1). As the age of piglets varied among samples, a linear mixed-effects model was fitted by setting the log_2_ neutralization titer as the response variable, the day-age of piglets sampled as the fixed effect, and the individual animals as the random effect.

Setting the simulated litters' neutralization titers as the initial values, the age-specific neutralization MDA titers were predicted up to the vaccination age according to the estimated slope, and the titers were then converted to S/P ratios.

### 2.6. Simulating the Immune Response to CSF Vaccination

The immune response of piglets against CSF vaccination depends on the level of MDAs at vaccination. To incorporate this difference in immune response into our simulation, we used the observed VIA S/P ratios for pigs on the vaccination day and at 4, 8, and 16 weeks postvaccination (Table 1). In our simulation, the dynamic change in the S/P ratio depending on the MDA level was resampled from the actual data and extrapolated in accordance with the agent's (i.e., the simulated animal's) MDA level at the time of vaccination.

The dynamics of the VIA S/P ratio after vaccination in the observed data were classified into four groups (low, medium, high, and very-high) according to the MDA S/P ratio (S/P < 0.2, 0.2 ≤ S/P < 0.4, 0.4 ≤ S/P < 0.6, and 0.6 ≤ S/P < 0.95, respectively) at the time of vaccination (*Supplementary 2*). For VIA traces in which the neutralization titer for the same individual declined to <2, the traces of the VIA S/P ratios were forced to decline to zero in the 16 weeks after the vaccination to avoid the artificial overestimate due to the flat slope. In each group, sets of slopes traced from individual pigs (0–4, 4–8, 8–16 weeks after vaccination) were pooled. In the simulation, (i) simulated agents at the vaccination age were typed by the MDA group, and (ii) a set of slopes was randomly selected from its MDA group, which represented the antibody dynamics until 16 weeks after vaccination. Although finisher pigs are slaughtered at 26 weeks of age, the slopes could not be extrapolated to the end because of the lack of data after 16 weeks postvaccination. We assumed that the predicted antibody titer at 16 weeks after vaccination remained constant up to 26 weeks. By observation, the slopes of the ELISA antibody titers were complex, and we decided not to introduce parametric assumptions on them. According to the previous study conducted in a Japanese farm that vaccine efficacy continues for 2 years; the assumption of constant antibody titer may be plausible [14]. MDAs with S/P ratios ≥ 0.95 at the age of vaccination were assumed to completely negate the vaccine's efficacy, and vaccination was modeled to make no changes in the decline in MDAs based on the previous observations [14]. For sow candidate agents, an immune response to the second and subsequent vaccinations was assumed to occur only in those agents not having acquired immunity (S/P ratio < 0.05) on the dates of vaccination. Otherwise, the antibody titer of sows was assumed to be constant after these vaccinations.

A simulation of 30 randomly selected sows that predicted the immune response of their litters on a farm was iterated 100 times, and the results were used for subsequent analyses.

### 2.7. Assessment of Herd Immunity Level

Herd immunity was evaluated using two criteria: (i) MDA-related protection against infection prior to vaccination and (ii) VIA-related protection. To evaluate MDA-related protection, MDAs with a neutralization titer of ≥6 log_2_ were assumed to offer protection based on experimental results from South Korea [20]. Using receiver operating characteristic (ROC) analysis with the R package ROCR [21], the cutoff value of the S/P ratio corresponding to 6 log_2_ neutralization titer was estimated to be 0.513 using S/P ratios and neutralization titers for all types of pigs (*n* = 1,021) sampled in Gifu Prefecture between April 2020 and March 2021 (Table 1). ROC analysis was used instead of the interconversion model to calculate the cutoff value because it can be directly calculated from the original data.

VIA protection was evaluated based on the S/P ratio at the slaughter of agents. A VIA S/P ratio ≥ 0.05 at slaughter was regarded as indicative of effective VIAs. The reason for using this method was that individual animals in which the antibody titer increases after vaccination are assumed to be immunized, regardless of the time it takes for the antibody titer to increase after vaccination. Agents with effective VIAs were assumed to have acquired immunity from 1 week after vaccination based on the previous studies [14, 22]. Age-specific herd immunity levels were represented by the proportions of pigs with either MDAs or VIAs that fulfilled the criteria.

### 2.8. Evaluation of the Optimal Vaccination Age and Vaccination Scenarios for G_2_ Agents

Six different options in terms of vaccination age, covering 4–9 weeks of age, were compared. The optimal age of vaccination for G_2_ fattening pigs was selected based on the proportion protected at the time of shipment to the slaughterhouse (26 weeks). The best vaccination age in this study was defined as the earliest week of age at which >70% of pigs would have protective immunity at slaughter. Regarding the proportion of pigs with protective immunity, 70% was used because in most of the scenarios in this model, the predicted proportion of pigs immunized did not reach 80%, which is recommended by the government [13].

The age-specific proportion of fattening pigs with protective immunity was compared between three scenarios: (i) a single shot of vaccine, (ii) adding a second vaccination at 1 month after the first vaccination, or (iii) adding a second vaccination at 2 months after the first vaccination. In each scenario with two shots, the first vaccination was fixed at 4 weeks of age, when the proportion of pigs with protective MDAs was predicted to be <70%.

### 2.9. Simulation of Antibody Titer Distribution in Sows and the Optimal Vaccination Ages for Their Litters that Change over Time

The high variation in antibody titers among sows due to a mixture of G_1_ and subsequent generations is a critical issue because that makes it difficult to maintain both the high proportion of pigs with effective MDAs and VIAs. To examine this problem, the dynamics in antibody titer distribution in sows after vaccination with a typical annual sow replacement rate was simulated every 6 months after the start of vaccination. The vaccination ages of litters born from the sows replaced every 6 months were then explored.

As addressed earlier, the replacement of sows was modeled as an event at 6-month intervals beginning 1 year after starting vaccination. Sows to be replaced at a 40% typical annual sow replacement rate were randomly selected from G_1_ sows. The selected sows were replaced with the same number of candidate sows that were randomly selected from among 1-year-old agents. After G_1_ sows were completely removed from the herd, the oldest sows were replaced. As none of the G_1_ sows remained at 3.5 years from the start of vaccination, replacement of sows was simulated until 4 years to monitor the subsequent 6-month period.

The best vaccination ages were explored for litters born from sows replaced every 6 months by comparing the calculated proportions of pigs with effective VIAs according to vaccination age. The candidate sows were assumed to be vaccinated at the best vaccination age and additionally vaccinated 6 months later.

### 2.10. Evaluation of Intensive Sow Replacement Policies

This study considered the effect of intensive sow replacement strategies. Three intensive replacement policies were evaluated besides the 40% (default) replacement rate. First, random selection with a replacement rate of 60% was examined. Second, the effect of preferential culling of G_1_ sows with an S/P ratio > 0.95 and a replacement rate of 60% was examined under the assumption that all G_1_ sows were tested for antibody titer. In this scenario, after the removal of G_1_ sows with an S/P ratio > 0.95, the remaining G_1_ sows were preferentially replaced. Third, in addition to the preferential culling of G_1_ sows with an S/P ratio > 0.95, we also assessed the effect of selecting candidate pigs with an S/P ratio between 0.1 and 0.95.

### 2.11. Validation of the Simulation Model

The validity of the model was evaluated by comparing the simulation results and the observed data from individual tracking ELISA results for fattening pigs. The comparison was conducted for fattening pigs that were vaccinated at 6 weeks old and slaughtered at 26 weeks old. Due to the limited available data, we targeted pigs that were slaughtered within 2 years after the first vaccination on a farm. The required sample size (*n*) was calculated in order to compare two proportions (i.e., simulation and observed) of protected pigs at slaughter age using Equation (4) [23]:

(4) $$\begin{array}{c} n={\left(1.96\sqrt{2p\left(1-p\right)}-0.84\sqrt{p_{1}\left(1-p_{1}\right)+p_{2}\left(1-p_{2}\right)}\right)}^{2}/{\left(p_{1}|-|p_{2}\right)}^{2}, \end{array}$$

where *p*_2_=*p*_1_ − 0.05, *p*=(*p*_1_ ± *p*_2_)/2.

In the above equations, *p*_1_ represents the proportion of fattening pigs with an S/P ratio ≥ 0.05 at slaughter, and *p*_2_ represents the marginal proportion of protected pigs at slaughter simulated by the model, allowing for a 5% difference from the field data. We calculated the sample size for pigs that were vaccinated at 6 weeks of age. The proportion of pigs vaccinated at 6 weeks of age with an S/P ratio ≥ 0.05 at slaughter (*p*_1_) was 66.7% (*n* = 30). The calculated sample size was 232, and test statistics were calculated for 250 of the randomly sampled simulated and observed results. The chi-square test was used to examine the difference in the proportion of pigs protected by MDAs (neutralizing titer ≥ 6 log_2_) at 6 weeks (at vaccination) and by VIAs (S/P ratio ≥ 0.05) at 26 weeks (at slaughter). The means of the S/P ratios from the simulated results and observed data at 6 and 26 weeks were compared using the Wilcoxon rank-sum test. The tests were performed 100 times to get 100 simulation results, with the significance level set at 0.05. R version 4.1.1 was used for all statistical analyses [24].

## 3. Results

### 3.1. Parameter Estimates for the Model to Predict MDA Decline

The parameters log_2_*NT*_*c*_, *a*, and *β* in the model to interconvert between S/P ratio and neutralization titer were estimated as 10.20 (95% confidence interval (CI): 9.43–10.96), 0.512 (95% CI: 0.418–0.571), and −3.770 (95% CI: −3.985 to −3.583), respectively. The neutralization titers converted from S/P ratios did not differ significantly from the observed neutralization titers (*p*=0.488, Kolmogorov–Smirnov test; *Supplementary 3*). The slope of the decline in the logarithm of neutralizing MDA titer was estimated as −0.097 (95% CI: −0.101 to −0.092) per day, and the calculated half-life was 10.31 days.

### 3.2. Simulated Results for Antibody Response Following Vaccination among Fattening Pigs

#### 3.2.1. Vaccination Age of G_2_

**Figure 4:**
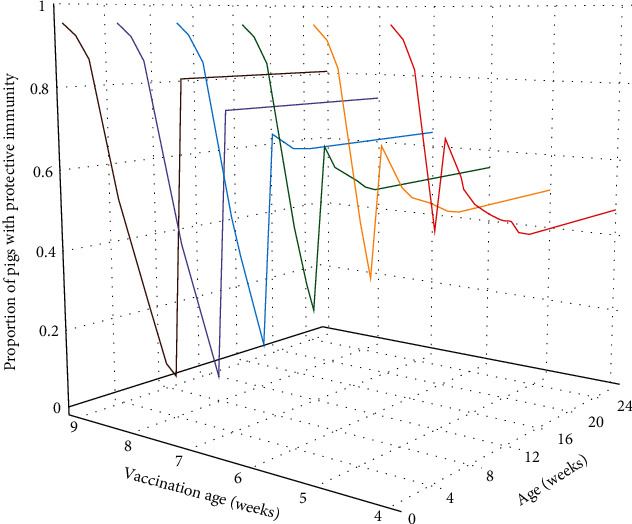
Age-specific proportions of G_2_ pigs with protective immunity at different vaccination ages (4–9 weeks) from birth to slaughter (26 weeks). Lines represent the means of the proportions.

Figure 4 shows the variation among the means of age-specific proportions of immune-protected G_2_ fattening pigs that were vaccinated at different ages (4–9 weeks of age). Eight weeks of age was determined to be the optimal age for vaccination, so that vaccination at that age was the earliest for achieving the required herd immunity threshold (i.e., 70%).

#### 3.2.2. Effect of Vaccinating Piglets Twice

**Figure 5:**
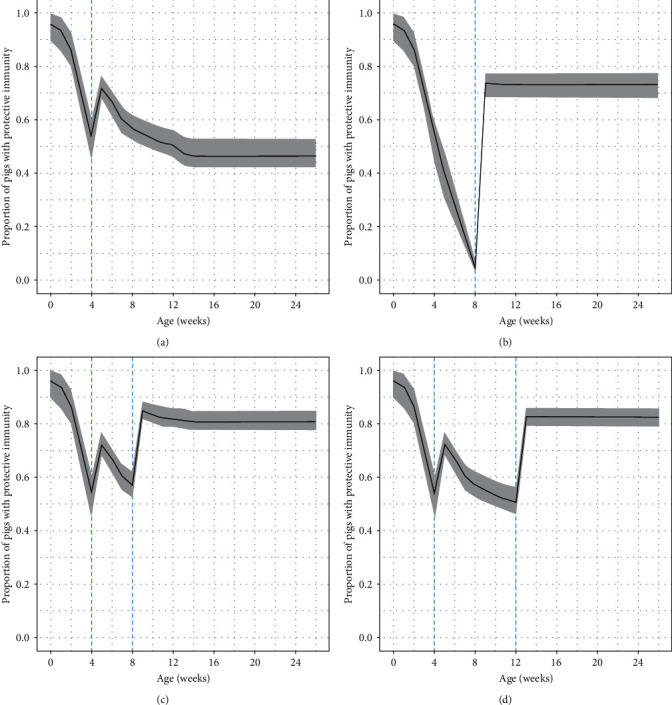
Age-specific proportion of G_2_ fattening pigs exhibiting protective immunity when vaccinated at (a) 4 weeks or (b) 8 weeks of age only, or 4 weeks of age with a second vaccination interval of (c) 4 weeks or (d) 8 weeks. Solid lines show the median, and ribbons indicate the 90% CI.

Figure 5 shows the difference in the age-specific proportions of G_2_ fattening pigs with protective immunity among the scenarios involving a different number of vaccinations and different vaccination ages (4 weeks only, 8 weeks only, 4 weeks and 8 weeks, 4 weeks and 12 weeks), with 90% CI. Vaccination at 4 weeks only resulted in a low proportion of pigs exhibiting an immune response (46.4% (90% CI: 42.2–52.8)) (Figure 5a), whereas vaccination at 8 weeks only resulted in a significantly low proportion of protection at the time of vaccination (4.4% (90% CI: 2.8– 6.9), Figure 5(b)). With regard to introducing a policy of vaccinating twice, the proportions of protection at slaughter were 80.6% (90% CI: 77.5%–84.4%) and 82.2% (90% CI: 78.6%–85.6%) for the 4- and 8-weeks intervals, respectively, both above 80% (Figures 5(c) and 5(d)). Whereas, in the weaning period, the 4-week interval resulted in a shorter period with low proportion of protection than the 8-week interval.

### 3.3. Simulation Results for Antibody Titer Transition after Starting CSF Vaccination

#### 3.3.1. Under an Annual Sow Replacement Rate of 40%

**Figure 6:**
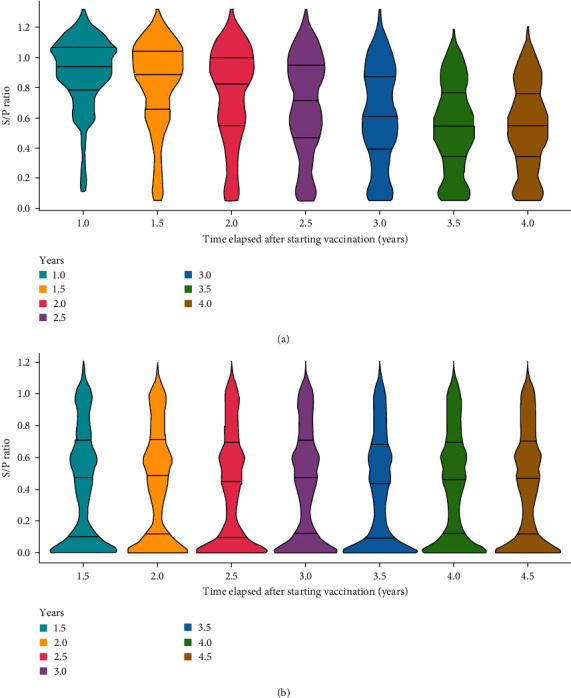
Distributions of S/P ratios for (a) sows (*n* = 3,000) and (b) fattening pigs at slaughter (*n* = 36,000). The vaccination age for fattening pigs was set at 8, 8, 7, 7, 6, 6, and 6 weeks of age in 0.5-year increments after the start of vaccination.

Figure 6 shows the progression of S/P ratios for (a) all sows (*n* = 3,000; a total of 100 iterations) and (b) fattening pigs at slaughter age born from these sows (*n* = 36,000; a total of 100 iterations) in a herd. Soon after the start of vaccination, the distribution of S/P ratios in sows was predicted to skew toward the high-titer side but then gradually shift toward the lower-titer side until 3.5 years after the start of vaccination. Then, the distribution would remain stable, with a median value of 0.535 (Figure 6(a)). The best vaccination age soon after the start of vaccination was 8 weeks old and shifted earlier to 7 weeks old after 2.0 years and then 6 weeks old after 3.0 years after the start of vaccination.

#### 3.3.2. Under Intensive Sow Replacement Policies

**Figure 7:**
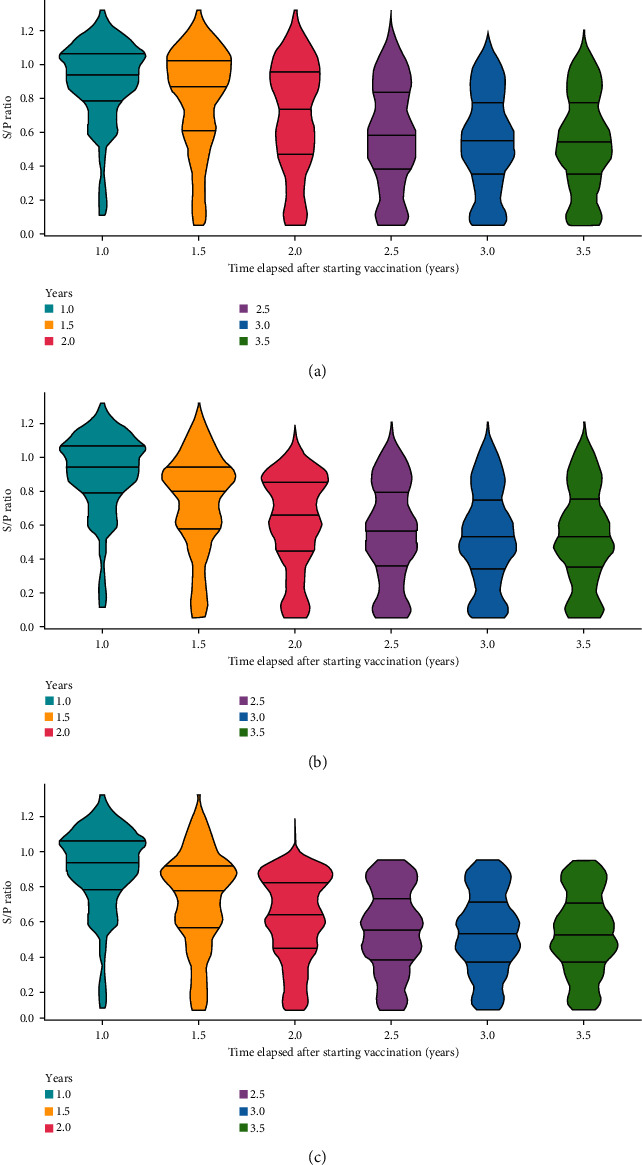
Distributions of S/P ratios of sow herds with (a) an annual sow replacement rate of 60%, (b) replacement rate of 60% and preferential culling of sows with an S/P ratio > 0.95, and (c) a replacement rate of 60%, preferential culling of sows with an S/P ratio > 0.95, and selection of candidates with 0.1 < S/P ratio < 0.95 in 0.5-year increments after the start of vaccination.

Figure 7 shows the shift in the S/P ratio distribution of sows at an annual sow replacement rate of 60%, (i) with random selection of G_1_ sows (Figure 7(a)), (ii) with preferential culling of G_1_ sows with an S/P ratio > 0.95 (Figure 7(b)), and (iii) with preferential culling plus selection of candidate pigs with 0.1 < S/P ratio < 0.95 (Figure 7(c)). The distribution of S/P ratios with a 60% replacement rate stabilized at 3.0 years (Figure 7(a)), which was 0.5 years earlier than the distribution with a 40% replacement rate (Figure 6(a)). By preferential culling, sows with an extremely high S/P ratio were eliminated at 2.0 years and stabilized at 2.5 years (Figure 7(b)), showing a phase transition 1 year earlier than that under the default replacement rate of 40% (Figure 6(a)). The scenario involving the selection of candidate sows with 0.1 < S/P ratio < 0.95 exhibited an increased proportion of pigs with an antibody titer near the median at 2.5 years (Figure 7(c)).

### 3.4. Validation of Simulation Model

The distributions of observed and predicted S/P ratios of fattening pigs were similar at 6 and 26 weeks (*Supplementary S4*). The predicted mean S/P ratios at 6 and 26 weeks (median = 0.318 and 0.317, respectively) were lower than the observed ratios (median = 0.375 and 0.392, respectively, median *p*-value = 0.057 and 0.001); however, no significant differences in the observed and predicted proportions of protected pigs were observed at 6 or 26 weeks (median *p*=0.502 and 0.329, respectively, Table 2).

## 4. Discussion

Following the resumption of CSF vaccination on swine farms in Japan in 2019 after the complete ban on CSF vaccination in 2006, higher antibody titers among sows were noted. This interfered with immunization by vaccination in young pigs, and intensive discussions on vaccination policy were initiated in an effort to overcome the difficulty of protecting pigs from CSFV. An epidemiologic investigation reported that CSF vaccination was highly effective in protecting pigs in farms [12]. The present study was designed to support data-driven discussions involving all stakeholders with regard to formulating CSF vaccination policies. The simulation results of the present study incorporated several critical points that stakeholders should consider in developing a CSF vaccination policy.

The first and the most important point for policy development relative to the dynamic change in antibody titers of sows after starting vaccination is the selection of the optimal vaccination age for piglets. This study demonstrated the importance of avoiding MDA-mediated interference with the immune response and proposed to determine the appropriate vaccination age based on the proportion of pigs with effective VIAs. The present results revealed that the earlier the vaccination age, the higher the proportion of piglets protected by MDAs at the time of vaccination, but the lower the proportion of piglets protected after vaccination, and vice versa.

The probability that MDAs will interfere with vaccination efficacy is high when CSF vaccination is given at an early age, particularly during the first year after the start of CSF vaccination on a farm. This study selected 8 weeks as the optimal vaccination age during the first year after initiating CSF vaccination. Our simulation results indicate that vaccination should be gradually shifted to a younger age as time elapses. Our results demonstrated that the optimal age reaches 6 weeks old, which is within the recommended range in a previous epidemic (35–42 days old) [14], at 3 years after starting vaccination.

The second important point with regard to policy development is the effect of intensive sow replacement strategies. Our results demonstrated that sows exhibit marked variation in antibody levels 1.5 years after the start of CSF vaccination due to the mixing of different generations of sows. As the MDA titer of piglets depends on their sow's antibody titer at delivery, the variation in MDAs in piglets is also high. This increases the difficulty of determining the optimal age of vaccination. Therefore, it is critically important to remove G_1_ sows with high titers. This study clearly showed the effect of intensive sow replacement on shortening the period with sows with diverse titers. Also, the option to replace sows with candidates with an S/P ratio in the medium range (between 0.1 and 0.95) can narrow the distribution of antibody titers among sows. Our simulation suggests that these policies would be effective in maintaining a narrow range of antibody distribution among piglets at a fixed age of vaccination. As preferential culling requires measurement of the titer of antibodies against CSFV among sows, these strategies should be considered with respect to cost-effectiveness.

The third point relevant to policy development is the effect of vaccinating weaning pigs twice. This option is designed to reduce the high risk of CSFV infection during the weaning period, during which there is a window of low immunization in piglets. The simulation results showed that a single vaccination at 8 weeks of age resulted in an extremely low proportion of piglets protected by MDAs. This issue was resolved by administering two shots at 4 and 8 weeks of age, as the proportion of piglets protected in the weaning period remained high, and the proportion of piglets with effective VIAs was >80%. However, the cost of vaccination and required labor are doubled in this case. In South Korea, in accordance with recommendations by the government, pig farmers give pigs one or two shots of attenuated live CSF vaccine [20]. The number of CSF outbreaks gradually declined after the introduction of the revaccination policy in 2003 [25]. The option of allowing farmers to select one or two shots should be considered. Analyses of feasibility and cost-effectiveness, as well as discussions among stakeholders, are needed. This option may be particularly useful during the period before a stable titer distribution in sows is achieved, as both the proportion of pigs with effective MDAs and VIAs can be kept high by vaccinating twice.

Some limitations in the present study should be mentioned. First, in this simulation study, results of ELISAs conducted at the time of slaughter were used to determine the appropriate age for vaccination. However, it has been suggested that there are pigs with effective neutralization titers among animals with S/P ratios < 0.05 by ELISA [26]. If the neutralization titer is used to determine whether immunity has been acquired, the calculated proportion of pigs immunized would be higher than our model predicted. As a result, the age of vaccination could be set earlier than the results of the present study indicated, which would reduce the risk to weaning pigs. Second, interconversion between the S/P ratio and neutralization titer allowed the prediction of the decline in MDAs based on their half-life on the neutralization titer scale. Nevertheless, the accuracy of the S/P ratio to neutralization titer conversion model is low with high S/P ratios. This problem was overcome by applying bootstrapping of actual neutralization titer values, but uncertainty remains as to whether such actual values provide a good prediction for samples with high CSF antibody titers. Third, the number of serological field test results for the same individual animals traced is still limited; therefore, future work to integrate more data could enhance the validity of the model. Furthermore, the slopes of antibody titers obtained from G_2_ piglets after their first vaccination were used to simulate the dynamics of antibody titer after the second and subsequent vaccination, as post–second vaccination antibody trace data were not available. Fourth, this study assumed that an MDA titer of ≥6 log_2_ provides full protection against virulent CSFV, as recently reported [20]. The CSFV strain prevalent in Japan in the 1960s and that used in the GPE^−^ vaccine both belong to genotype 1 [10], but the strain currently circulating in Japan belongs to genotype 2.1 [9]. The level of MDAs that provides protection against the current field strain and the associated S/P ratio as determined using the currently available ELISA remain unknown. Experiments to clarify these parameters are needed. Fifth, it has been reported that infection with porcine reproductive and respiratory syndrome virus or porcine circovirus type 2 interferes with the immunological response to CSF vaccines [27–29]. The present simulation model did not take this into account; therefore, the immune response in this model may be overestimated for farms with a high prevalence of these infections.

## 5. Conclusion

This study clarified the risk of CSFV infection during the weaning period and the need to select the optimal vaccination age according to the antibody titer of a herd at that time. This study demonstrated that when CSF vaccination is initiated in naïve swine herds, it is necessary to delay the vaccination age compared with immunized herds and gradually shift the vaccination to younger ages. The simulation results indicated that the risk of CSF infection is higher during the weaning period on farms using GPE^−^ vaccines.

This study described an evidence-based approach for selecting the appropriate age for vaccination after initiating a vaccination program. Our results suggest that several years are required to achieve a stable antibody titer distribution among sows; however, this period can be shortened by intensive sow replacement. The risk in weaning pigs can be reduced by vaccinating twice. In addition to the issues addressed in the present study, the strengthening of biosecurity measures should also be addressed.

## Figures and Tables

**Table 1:** Purpose, type, number, and source of monitoring data used for modeling.

Purpose	Type of pigs (sample size)	Source
1. Estimate model parameters for interconverting neutralization titers and S/P ratios	All types of pigs with neutralization titer ≤ 10 log_2_ (*n* = 1,160 for parameter estimation and *n* = 1,181 for model validation)	S/P ratios and neutralization titers of pigs on seven farms sampled between May 2020 and December 2021
2. Predict neutralization titers of 1-day-old piglets from neutralization titers of their sows by resampling	Sows (*n* = 9) and their litters (*n* = 63)	Neutralization titers of pigs in two farms sampled between May 2020 and September 2021
3. Predict decline in neutralization titers of MDAs among piglets	Piglets before vaccination (*n* = 387)	Neutralization titers of pigs on seven farms sampled between May 2020 and December 2021
4. Sample the S/P ratio distribution of G_1_ vaccinated sows	G_1_ sows (*n* = 168)	S/P ratios of G_1_ sows in Gifu Prefecture sampled between April and July 2020
5. Predict changes in VIA levels among fattening pigs	G_2_ fattening pigs with low, medium, high, and very-high MDA levels at the age of first vaccination (*n* = 26, 29, 24, and 11, respectively)	S/P ratios of G_2_ fattening pigs in Aichi and Gifu Prefectures sampled between June and October 2020
6. Calculate S/P ratio cutoff values for log_2_ neutralization titers	All types of pigs from farms (*n* = 1,021)	S/P ratios and neutralization titers of pigs in Gifu Prefecture sampled between April 2020 and March 2021
7. Validate the prediction of antibody titers among fattening pigs	Fattening pigs at 6 and 26 weeks in a farm 2 years after the start of vaccination (*n* = 30)	S/P ratios of fattening pigs vaccinated at 6 weeks on a farm in Gifu Prefecture sampled between May and October 2021

**Table 2:** Comparison of observed and predicted S/P ratio distributions and proportions of pigs protected by MDAs (neutralizing titer ≥ 6 log_2_) or VIAs (S/P ratio ≥ 0.05) and results of model validation by Wilcoxon rank-sum test and chi-square test.

Age (weeks)	Mean S/P ratio 50th percentile (5th and 95th percentiles)		Proportion of protected pigs 50th percentile (5th and 95th percentiles)		*p*-Value 50th percentile (25th and 75th percentiles)	
	Observed	Predicted	Observed	Predicted	Wilcoxon rank-sum test	Chi-square test
6^a^	0.375 (0.345, 0.396)	0.318 (0.286, 0.343)	0.204 (0.152, 0.232)	0.208 (0.144, 0.256)	0.057 (0.007, 0.288)	0.502 (0.202, 0.826)
26^b^	0.392 (0.349, 0.433)	0.317 (0.275, 0.365)	0.664 (0.604, 0.716)	0.624 (0.560, 0.680)	0.001 (<0.001,0.009)	0.329 (0.119, 0.646)

^a^At the time of vaccination; all S/P ratios are MDAs. ^b^At the time of slaughter; S/P ratios are VIAs.

## Data Availability

This study used national and prefectural governmental data, and access to the data can be considered upon request to the authors.

## Supplementary Materials

Supplementary 1 Relationship between neutralization titer and S/P ratio for all types of pigs at seven farms (empty circles, n = 1,181).

Supplementary 2 Observed changes in S/P ratios after vaccination, classified into four groups according to the S/P ratio at the age of vaccination.

Supplementary 3 Comparison of converted and observed neutralization titers.

Supplementary 4 Distributions of observed and predicted S/P ratios of fattening pigs (a) at 6 weeks (at vaccination) and (b) at 26 weeks (at slaughter) of age, 2 years after the start of vaccination on the farm (n = 25,000 resampled S/P ratios).
