# Supplementary figures and images for: Exploring Appropriate Strategies for Vaccination against Classical Swine Fever under a Dynamic Change in Antibody Titer in Sows after Starting Vaccination in a Japanese Farm Setting
Source: Transbound Emerg Dis. 2023 Nov 30;2023:5541976. doi: 10.1155/2023/5541976 (PMC12016941; doi:10.1155/2023/5541976)

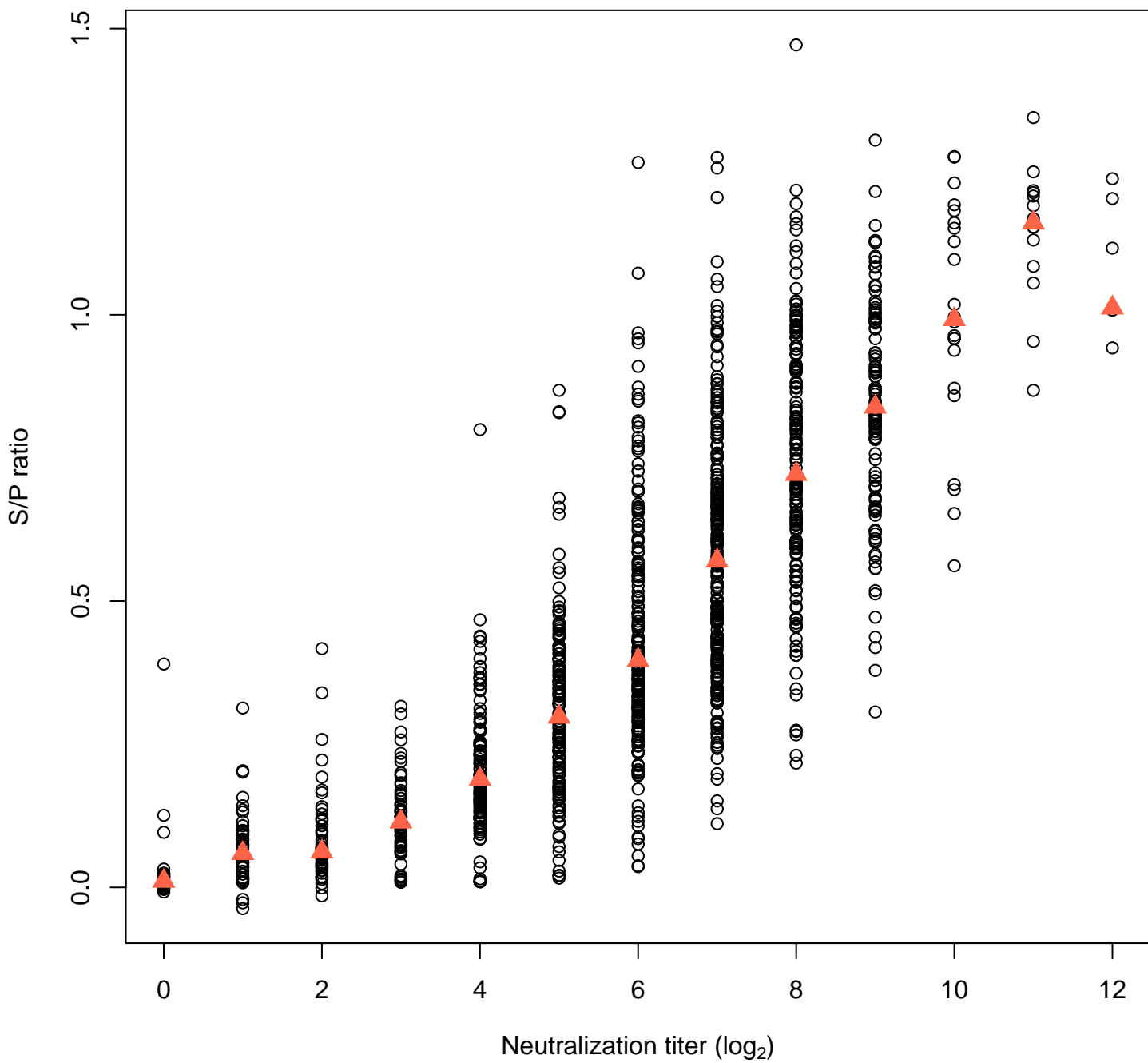

Supplement: Supplementary 1 — Relationship between neutralization titer and S/P ratio for all types of pigs at seven farms (empty circles, n = 1,181). [file 5541976.f1.pdf]

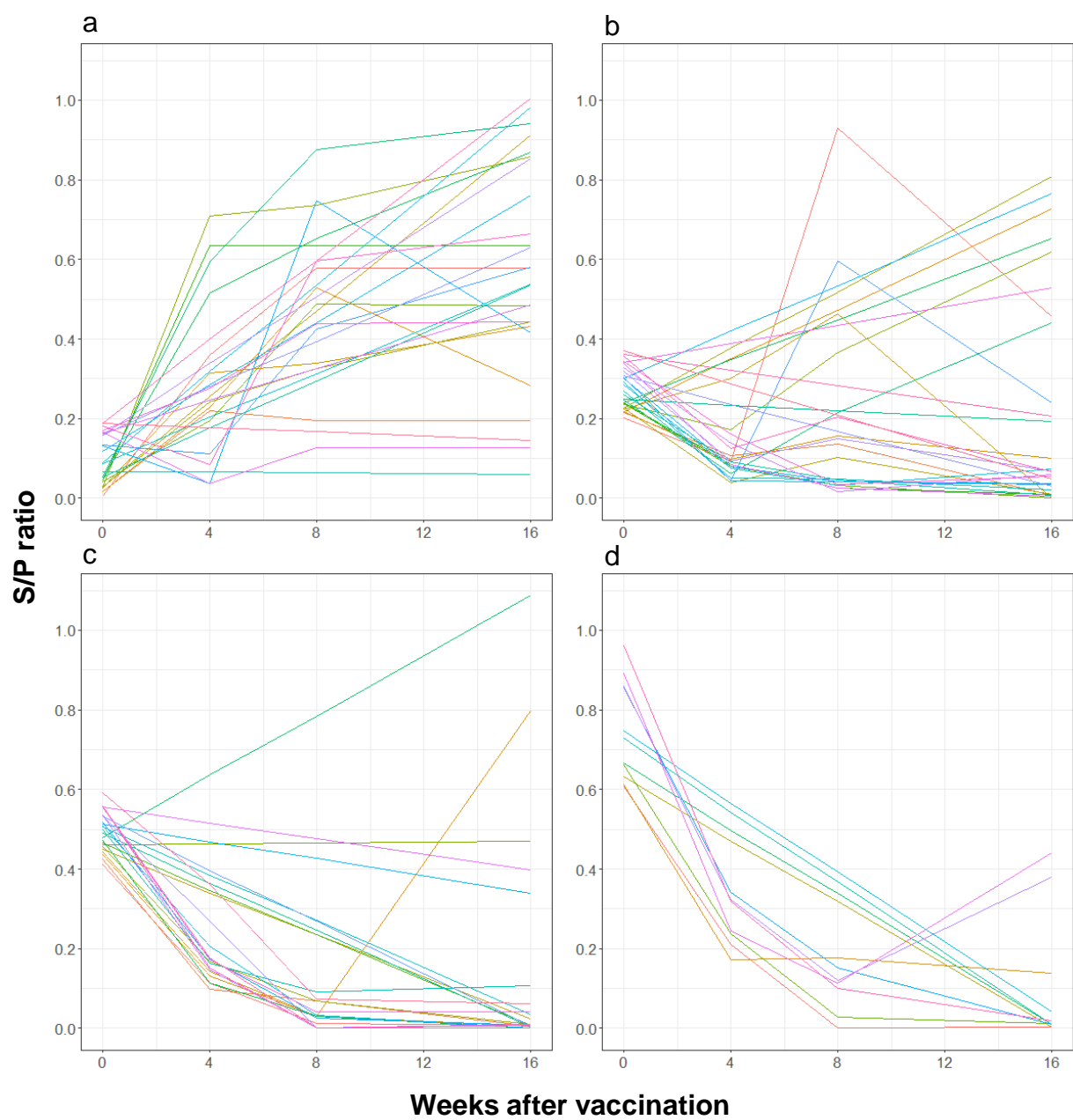

Supplement: Supplementary 2 — Observed changes in S/P ratios after vaccination, classified into four groups according to the S/P ratio at the age of vaccination. [file 5541976.f2.pdf]

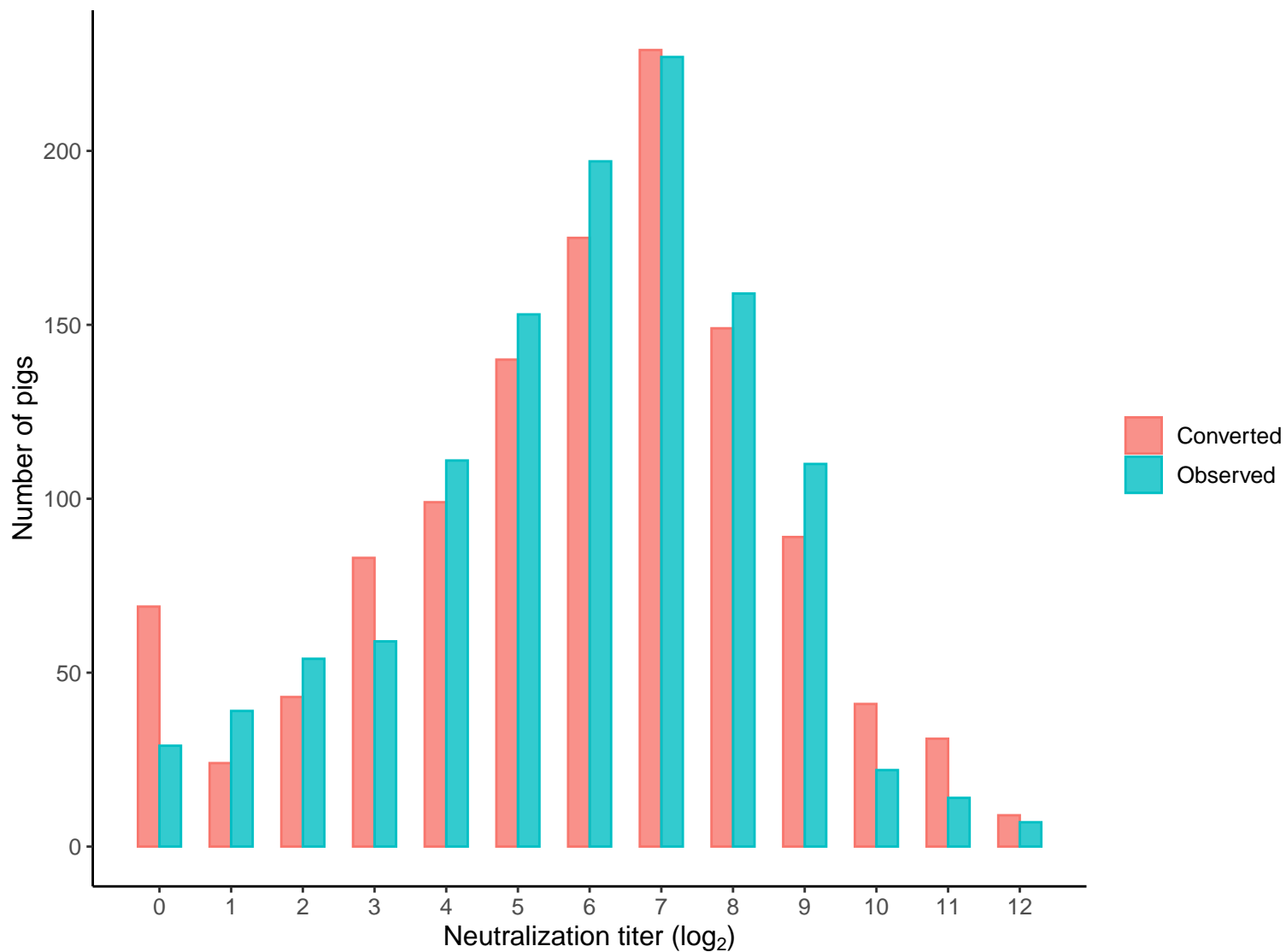

Supplement: Supplementary 3 — Comparison of converted and observed neutralization titers. [file 5541976.f3.pdf]

**a**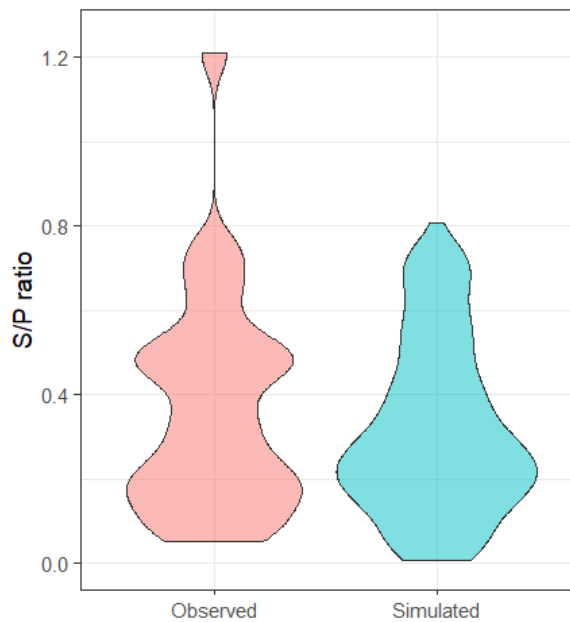**b**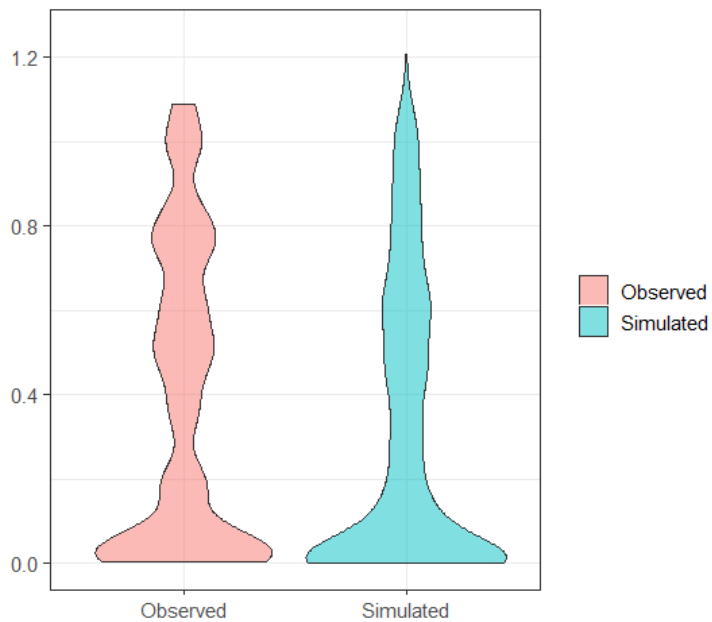

Supplement: Supplementary 4 — Distributions of observed and predicted S/P ratios of fattening pigs (a) at 6 weeks (at vaccination) and (b) at 26 weeks (at slaughter) of age, 2 years after the start of vaccination on the farm (n = 25,000 resampled S/P ratios). [file 5541976.f4.pdf]
